# Aesthetic Comparison between Subcuticular Suture and Staple Closure of Anterior Cervical Decompression and Fusion Incision Scars: A Prospective Controlled Single-blinded Trial

**DOI:** 10.7759/cureus.3996

**Published:** 2019-02-01

**Authors:** Sina Rajamand, Diana Kakos, Doris Tong, Lucas Garmo, Teck M Soo

**Affiliations:** 1 Neurosurgery, Ascension Providence Hospital Michigan State University College of Human Medicine, Southfield, USA; 2 Internal medicine, Wayne State University School of Medicine, Bloomfield Hills, USA

**Keywords:** anterior cervical incision, scar, cervical fusion, neck scar, wound closure

## Abstract

Introduction: Anterior cervical decompression and fusion (ACDF) procedures are common and neck scar appearance is important aesthetically. This study compared subcuticular suture closure with staple closure regarding the aesthetics of the neck incision scar.

Methods: A single-blinded comparative prospective study with two cohorts involving one facility and multiple surgeons was done to study all consecutive patients who underwent one/two-level ACDF operations from 2015-2016. We excluded patients with corpectomies, postoperative wound infection, reoperations in the same admission, any previous ACDF operations, non-compliance in follow-up, and inability to give informed consent. We did single-layer skin stapling without platysma closure or subcuticular suture with platysma closure. Patients followed up between 1.5 and six months. We used the Stony Brook Scar Evaluation Scale (SBSES), range 0-5 with five being the best score. Digital images were taken in a standardized manner and saved in a secure database. A blinded plastic surgeon and a blinded neurosurgeon, not involved in the operation, evaluated the scars using SBSES. A priori sample size using a clinically significant difference of one was determined. Wilcoxon rank-sum test was used; a p-value <0.05 was considered statistically significant.

Results: We studied 93 staple and 66 suture closures. There is no significant difference between the groups regarding age, sex, the incidence of diabetes, smoking, obesity (body mass index (BMI) >30 kg/m2), chemotherapy or the length of the incision. There is no statistically significant difference regarding SBSES as evaluated by the plastic surgeon (staples vs. sutures, median 2 vs. 2, range 0-5, p = 0.32). There is a statistically significant difference as evaluated by the neurosurgeon (staples vs. sutures, median 4 vs. 3, range 0-5, p = 0.003). Post hoc power analysis was 0.90.

Conclusion: Using the validated SBSES to assess the aesthetic outcome of ACDF scars, we demonstrated that staples and sutures provide equivalent aesthetic outcomes per plastic surgeon evaluation, and staple closure results in statistically significant better aesthetic outcomes per neurosurgeon evaluation.

## Introduction

Anterior cervical spine procedures make up a significant portion of the clinical caseloads of neurosurgeons and orthopedic spine surgeons [1]. It is also a relatively common procedure among the general population [2]. Any procedure of the head and neck will receive greater scrutiny of scar appearance, as the scar is readily apparent to the patient and observers.

An anterior cervical incision can be closed in many ways, two of which are sutures versus staples, with sutures being the more common technique. Patient satisfaction with these two wound closure techniques is comparable [3]. However, there is a lack of peer-reviewed literature comparing the aesthetic outcomes of the scar using the two different techniques [4]. We seek to demonstrate that staple closure in anterior cervical incision gives an equivalent aesthetic outcome when compared to subcuticular suture closure.

Our study conclusions will help surgeons make more informed decisions regarding wound closure technique, leading to better patient outcomes.

## Materials and methods

This is a prospective single-blinded comparative cohort study with two groups involving one facility and multiple surgeons. Our study was IRB approved for a duration of 15 consecutive months in 2015-2016, where we evaluated consecutive patients undergoing one or two-level anterior cervical decompression and fusion (ACDF) who met our inclusion criteria. Formal consent was obtained from individual patients during their preoperative visit. We excluded patients who underwent corpectomies, re-opening and re-closure of the cervical wound due to non-wound related complications, and previous cervical operations with an incision in the area. All consented patients were tracked and interviewed postoperatively between 1.5 and six months for the scar assessment. A plastic surgeon and a neurosurgeon, blinded to the type of closure technique rated the scars using the Stony Brook Scar Evaluation Scale (SBSES) [5]. The operating neurosurgeon was not involved in the scar assessment.

Staple closure entails no platysma closure and is comprised of one layer of skin closure using a stapler. The skin edges are everted and approximated using two Adson forceps and staples are placed across the incision line. Staples are placed approximately 0.5-1 cm apart for the full length of the incision. The staples are then optimally removed between seven-ten days after the surgery. However, due to poor compliance by some patients, they have been removed as late as 34 days with no hash marks and an overall aesthetically pleasing scar. Subcuticular suture closure technique involves closing the platysma with absorbable, braided 3-0 Vicryl suture (Ethicon Inc., Sommerville, NJ) followed by a running subcuticular 4-0 Monocryl (Ethicon Inc., Sommerville, NJ) monofilament absorbable suture in the standard fashion followed by the placement of Steri-Strips (3M, St. Paul, MN).

The validated SBSES, as seen in Table 1, is comprised of five categories. The Principal investigator reviewed the categories with the surgeons prior to the grading of the scar. The review process entailed assigning 0 (zero) points for the presence of, or one point for the absence of the following [5]: (1) a width greater than 2 mm at any point of the scar; (2) a raised or depressed scar in relation to the surrounding normal skin; (3) red, blue, brown, or black discoloration of the scar relative to the surrounding normal skin; (4) any hatch marks on either side of the scar from sutures or staples; and (5) overall poor or ugly appearance.

**Table 1:** Stony Brook Scar Evaluation Scale (SBSES)

Stony Brook Scar Evaluation Scale (SBSES)	Score
Width	
>2 mm	0
<2mm	1
Height	
Elevated or depressed in relation to surrounding skin	0
Flat	1
Color	
Darker than surrounding skin (red, purple, brown, black)	0
Same color or lighter than surrounding skin	1
Hatch marks or suture marks	
Present	0
Absent	1
Overall	
Poor	0
Good	1
Total Score = sum of individual scores: range, 0 (worst) to 5 (best)	

The scar assessment was performed through images. The use of images as the method of assessment was described in the validation study of the SBSES [5]. A picture of the surgical scar was obtained using the Lumix® DMC-SZ1 16.1 megapixel digital camera. The pictures were taken at 1.5, three, and six-month follow-up appointments. All photographs were obtained 12 inches away from the scar in a well-lit area designated for the study. The photographers were trained to produce consistent picture quality before the start of the study.

Our primary outcome is the SBSES score for the sutures and the staples closures. Each pair is matched by intervals. To determine if the stapling method is equivalent to the suture method, a Mann-Whitney rank sum test was used. Our secondary outcomes were patients’ subjective pain evaluation as measured by visual analogue scale (VAS) at each interval. These scores were analyzed using the Mann-Whitney rank sum test. A p-value less than 0.05 is considered significant.

Agreement between the plastic surgeon and the neurosurgeon was tested by intraclass correlation (ICC) using the absolute agreement definition and a two-way random effects model. Imbalance of confounding variables such as age, sex, length of the scar, and length of surgery was analyzed in a post-hoc-stratified analysis using the Mantel-Haenszel procedure. The p-value was adjusted for multiple comparisons.

Sample size calculations were based on the comparison of two means with a type one error of 0.05 and a power of 0.8. We used a value of 3.1 for group A (staples) to detect a clinically significant difference of one. We chose the values for the means in both groups and a clinically significant difference based on one of our reference articles [6], and we chose the group means which will require the most patients in each arm. The total sample size needed in the study was calculated to be 52 with 26 in each arm. Sensitivity analyses were performed when appropriate.

## Results

We studied 93 staple and 66 suture closures. Five patients whose scars were inaccessible due to hair growth or wrinkles were entered into a sensitivity analysis assuming the best and worst scores in each arm. No patients were lost to follow up. There was no wound infection, dehiscence, or other wound complications. There is no significant difference between the groups regarding age, sex, the rate of diabetes, smoking, obesity (body mass index (BMI) >30, chemotherapy, and length of incision (Tables 2-3). The ICC showed fair agreement between the plastic surgeon and the neurosurgeon, both blinded to the patients and to each other’s ratings (ICC p=0.552, 95% CI 0.071-0.755).

**Table 2:** Patient Demographics

Patient Demographics			
N= 159	Suture (n=66)	Staples (n=93)	p value
Gender (% Male)	47%	50.5%	0.66
Age (Mean +/- SD)	52.65 ± 12.21	52.19 ± 13.23	0.82
BMI > 30 (%)	40	34	0.52
Diabetes (%)	17	16	0.92
Smoking (%)	24	32	0.27

**Table 3:** Peri-operative and Scar Variables

Peri-operative and Scar Variables			
N=159	Sutures (n=66)	Staples (n=93)	p value
Length of Scar (mm)	46.44 +/- 12.16	45.97 +/- 12.77	0.82
Length of surgery (min)	77.34 +/- 27.35	74.98 +/- 30.41	0.64

The neurosurgeon found a statistically significant better aesthetic outcome for staple closure when compared with suture closure (median 4 vs. 3, range 0-5, p=0.003) while the plastic surgeon found no difference between staples vs. sutures (median 2 vs. 2, range 0-5, p=0.331) (Table 4).

**Table 4:** Stony Brook Scar Evaluation Scale (SBSES) Scoring by Plastic Surgeon and Neurosurgeon

SBSES – Scoring by Plastic Surgeon and Neurosurgeon			
N=159	Sutures (n=66)	Staples (n=93)	p value
Plastic Surgeon (median/range)	2/0-5	2/0-5	0.331
Neurosurgeon (median/range)	3/0-5	4/0-5	0.003

VAS scores for wound pain showed no significant difference between the groups (P=0.11). Post hoc power analysis showed 0.90.

## Discussion

To our knowledge, there are no studies to date comparing closure of anterior cervical spine incisions using sutures vs. staples. Both manners of closure are fairly quick; however, the staples closure is faster and more efficient [3,7]. Suture closure is performer dependent, with technique playing an imperative role in the final outcome of the scar, whereas staple closure provides a much more uniform closure technique. However, stapling requires two operators with one everting the skin edges whereas suturing can be done by a single operator.

We have also found that in the rare cases of airway compromise due to hematoma formation, staples are much more easily and quickly removed for hematoma evacuation than sutures would be. The incision is also closed again with staples more easily after hematoma evacuation due to less trauma to the skin edges than the removal and realignment with needle and thread. Another advantage of staples is that each individual staple aligns a short portion of the incision, whereas the entire incision is dependent on one continuous subcuticular suture in a suture technique. If the suture is compromised in any way the entire incision has the potential of unraveling.

Our study limitations include the use of one neurosurgeon and one plastic surgeon to analyze the aesthetic outcome of sutures versus staple closure. Future studies should include more judges with a larger population utilizing a randomized control trial to further confirm our findings. Another limitation is stapling requires two operators with one everting the skin edges.

## Conclusions

Our study shows that the aesthetic outcome of the closure of an anterior cervical spine incision with staples to be at least equivalent or more superior to the standard closure technique using suture. Future studies should incorporate a larger sample size with more surgical wound assessors in order to strengthen inter-rater reliability. 
